# Multivariate analysis of independent roles of socioeconomic status, occupational physical activity, reproductive factors, and postmenopausal hormonal therapy in risk of breast cancer

**DOI:** 10.1007/s10549-022-06571-x

**Published:** 2022-04-02

**Authors:** Sushmita Katuwal, Juha Tapanainen, Eero Pukkala

**Affiliations:** 1 Faculty of Social Sciences, Tampere University, Arvo Ylpön katu 34, 33520 Tampere, Finland; 2 Department of Obstetrics and Gynecology, University of Helsinki, Helsinki University Hospital, Helsinki, Finland; 3 Department of Obstetrics and Gynecology, PEDEGO Research Unit, Medical Research Center, Oulu University Hospital, University of Oulu, Oulu, Finland; 4 Finnish Cancer Registry, Institute for Statistical and Epidemiological Cancer Research, Helsinki, Finland

**Keywords:** Breast cancer, Etiology, Risk factors, Hormonal replacement therapy, Socio-economic status, Physical activity, Parity

## Abstract

**Purpose:**

This case–control study assesses the independent roles of reproductive history, postmenopausal hormonal therapy (HT), socioeconomic status (SES), and occupational physical activity on the risk of breast cancer (BC).

**Methods:**

Odds ratios (OR) were estimated from conditional logistic multivariate regression model in a data set of 19,253 Finnish women diagnosed with BC between 1994 and 2013 and 96,265 age-matched population controls.

**Results:**

Both pre- and postmenopausal white-collar workers had significantly increased risk of ductal and lobular BC as compared to manual workers. Moderate occupational physical activity reduced risk of lobular BC by 14%. There was a transient increase in the risk of BC observed after each birth followed by a protective effect starting some years after the delivery. As the number of children increased, the short-term excess risk was lower and protective effect was observed earlier. Continuous estrogen-progestin therapy (EPT) significantly increased the risk of both ductal and lobular BC and the magnitude of risk was directly proportional to duration of use (OR for 5+ years of use 2.26, 95% confidence interval 2.12–2.42). Monthly EPT for 5+ years increased the risk (OR 1.32, 95% CI 1.20–1.45). Users of estradiol plus levonorgestrel intrauterine system devices showed ORs of 1.56 (95% CI 1.45–1.69) and 2.18 (95% CI 1.81–2.64) for ductal and lobular BC, respectively.

**Conclusion:**

This study concludes that pregnancy has a dual effect on BC risk, with a transient increase in risk followed by a long-term protective effect. The SES and HT have a large effect on BC risk while occupational physical activity has only a small independent effect.

**Supplementary Information:**

The online version contains supplementary material available at 10.1007/s10549-022-06571-x.

## Introduction

Breast cancer is the most common malignancy worldwide, with around 2.26 million new diagnosis among female estimated in the year 2020 [1] In Finland, breast cancer is the leading cancer diagnosis among women with the age-standardized incidence rate of 92 per 100,000 person-years in 2018, standardized to the World Standard Population [2].

Reproductive factors such as low parity and high age at first birth, lack of breastfeeding, early menarche and late menopause are well established risk factors for breast cancer in epidemiological and clinical studies [3–5]. The hormonal mechanisms involved in reproductive processes influence the breast cancer development, either by stimulating or inhibiting the factors that are responsible for initiation of breast cancer or its early growth [6–8]. Especially, endogenous estrogens play several roles in neoplastic transformation of breast tissue, either as carcinogenic agents or as permissive, promotional and tumor growth-inducing agents [9, 10]. In addition to indigenous hormones, exogeneous hormone therapy to manage menopausal symptoms are also implicated as risk factors in the development of breast cancer [11–13].

Recently there has been increasing interest to associations of work environment and breast cancer risk. Physical inactivity has shown to be associated with breast cancer, but the results concerning the occupational physical activity are inconsistent [14–18]. In addition, some characteristics of the modern work life such as career planning and demands of work may lead to postponement of childbirths and thus indirectly influence breast cancer risk [19–23].

The risk of breast cancer is determined by complex mechanisms involving individual’s genetic, physiological, reproductive, lifestyle and environmental factors. Numerous epidemiologic studies have examined the relation between breast cancer and single risk factors individually but there are not many publications on the independent roles of multiple factors in same study. Although it has been acknowledged that the breast cancer is not a single disease with a uniform etiology, the etiological differences in risk according to age at diagnosis and other characteristics of tumor such as histology are not adequately studied [5, 24]. The present population-based study was designed to explore the risk of breast cancer in a multifactorial setting that allows assessment of independent roles of numerous components of reproductive history, postmenopausal hormonal therapy (HT), socio-economic status (SES) and occupational history stratified according to characteristics of breast cancer such as histology and age at diagnosis.

## Materials and methods

### Study subjects

This is a retrospective case–control study, which includes all Finnish women who were diagnosed with their first breast cancer (ICD-10 code C50) between 1st January 1994 and 31st December 2013. Altogether 19,253 cases were identified in the national population-based Finnish Cancer Registry. The cancers recorded by the Cancer Registry have been notified by hospitals, pathological and hematological laboratories, physicians, and dentists, and from death certificates [25].

For each case of breast cancer, five female controls were randomly selected from the Finnish National Population Registry matched by the year of birth. The controls had to live in Finland at the time of cancer diagnosis of the case (index date). The Population Registry also provided information on the dates of birth of biological children of the cases and controls.

### Socioeconomic and occupational variables

Information about SES, education, and occupational history of all the study subjects was obtained from Statistics Finland. SES in our study is classified according to Statistics Finland’s classification of socioeconomic groups 1989, which is based on the classification of the United Nations Economic Commission for Europe (Appendix 1). The classification is formed by several criteria considering persons stage in life (family member, student, economically active, pensioner, etc.), occupational status, and nature of occupation [26]. For the purpose of our study, we categorized SES into upper-level white-collar employees, lower-level white-collar employees, manual workers and others as explained in Appendix 1. Educational levels were based national levels of education corresponding to the International Standard Classification of Education (ISCED 2011) of the United Nations Educational, Scientific and Cultural Organization.

### Occupational physical activity

For all study participants, information on occupational history was obtained from Statistics Finland. Finland's national classification of occupations is based on the international classification of occupations 2010 (ISCO-08) of the International Labour Organization (ILO). The national classification of occupation has been revised in 1987, 2001 and 2010. We used a conversion key for converting the occupational codes to the 311 categories of the longitudinal occupational classification used in the NOCCA Job Exposure Matrix (NOCCA-JEM; [27]). In the NOCCA-JEM, physical activity at work is expressed as perceived physical workload (PPWL), which is a quantitative amount given to each occupation based on perceived workload reported in quality of the Finnish Work Life Survey in 1990 [27]. The PPWL is characterized by probability of exposure (P) and average exposure level among those exposed (L) in each occupation. L is expressed in scale 0–1 where values near 1 are categorized as very heavy workload and value approaching 0 as sedentary work. Cumulative exposure to PPWL for each case and control was calculated by multiplying P*L values with the time (T) worked in that occupation from the age of 20 (typical starting age in most occupations) to age of 65 years or age at index date, whichever was lower. If an individual changed occupation between the censuses, she/he was assumed to have changed occupations in the middle of the period between the known census years. The cumulative PPWL exposure non-zero values were divided into mild (lowest 50%; < 0.26 PPWL years), moderate (values between the 50 and 90 percentile; 0.26–3.89 PPWL years) and high level of physical activity (highest 10%; 3.90–28.35 PPWL years). The number of women in mild physical activity was small and cumulative PPWL was very low, we therefore combined this category to sedentary occupations with P*L*T value as 0. We classified economically inactive women as a separate category. This category includes mostly housewives and farmers’ wives, majority of which are physically active. Therefore, the final categories for occupational physical activity were sedentary, moderate, high and economically inactive.

### Hormone therapy

Information on postmenopausal HT was obtained from the nationwide Prescription Registry of the Social Insurance Institution of Finland. The registry includes data on systemic HT purchases in Finland since 1994. Systemic HTs are available in Finland only with doctor’s prescription and automatically registered. In our study, purchases of HT at the age of ≥ 50 years for a minimum duration of 6 months was considered as postmenopausal HT.

Only estradiol (E) was used as the estrogen component during the study period. Systemic HT in our study was categories as E only, E combined with progestin therapy (EPT) and E (oral or transdermal) plus levonorgestrel-releasing intrauterine system (E+LNG-IUS). EPT was defined as continuous when oral or transdermal E was combined with daily progestin, as monthly when progestin was given for 10–14 days every month and once-in-3-months progestin when progestin was given for 10–14 days every 3 month. Information on the removal date of intrauterine devices is not available and therefore, the duration of LNG-IUS exposure was defined assuming that a woman who purchased one device used it for five years, which is the average duration of LNG-IUS use in Finland [28].

### Statistical analyses

A conditional logistic regression model was used for matched cases and controls for both univariate and multivariate analyses. Odds ratio (OR) with 95% confidence intervals (95% CI) was used to evaluate the association between study variables and breast cancer. We tested for the correlation between SES, education and occupational physical activity as well as fitted several alternate models with the combination of these variables. We dropped education variable from our final analysis because educational information was correlated with SES and was unknown for a large proportion of study participants.

Reproductive variables in our study are parity (categorized as nulliparous, parous); number of children (1, 2, 3, 4, 5+); age at first birth (< 20, 20–24, 25–29, 30+ years); age at last birth (< 30, 30–34, 35–39, 40+ years). Duration of use of each type of HT was categorized into < 1 year, 1–< 5 years, 5+ years; except for E+LNG-IUS which is categorized as number of devices purchased (1 and 2+ purchase). The analyses were stratified according to age at breast cancer diagnosis (< 50 years, called “premenopausal”; ≥ 50 years, “postmenopausal”), and by histology (ductal, lobular).

To estimate the dual effect of pregnancy on breast cancer risk, a conditional logistic regression was run with parity, time since delivery and interaction between these two variables included in the model. The model was adjusted for SES. The fitted results of this model were plotted for the breast cancer risk by parity and time since the delivery. All analyses were performed using R statistical software, version 1.2.1335.

## Results

Out of the 19,253 breast cancer cases, 82% were diagnosed at the age 50+ years, and 78% were of ductal and 16% of lobular subtype (Table 1).

**Table 1:** Characteristics of cases and controls

	Cases		Controls	
	*N*	%	*N*	%
All	19,253	100	96,265	100
*Histology*^a^				
Ductal	15,031	78	75,155	78
Lobular	3095	16	15,475	16
Other	1127	6	5635	6
*Age at diagnosis*^**a**^				
< 50 years	3388	18	16,940	18
50+ years	15,865	82	79,325	82
*Socio-economic status*				
Manual workers	3215	17	18,451	19
Lower-level employees	7489	39	35,917	37
Upper-level employees	3481	18	14,058	15
Others	5000	26	27,382	28
*Occupational physical activity*				
Sedentary	9460	49	44,687	46
Moderately active	6923	36	36,347	38
Highly active	1848	10	8977	9
Economically inactive	1022	5	6254	6
*Parity before index date*				
0	3463	18	15,406	16
1	3940	20	17,695	18
2	7317	38	36,378	38
3	3292	17	18,343	19
4	885	5	5739	6
5 +	356	2	2704	3
*Age at first birth*				
< 20 years	1928	12	10,972	13
20–24 years	6045	38	33,714	42
25–29 years	4819	31	23,498	29
30+ years	2998	19	12,672	16
*Age at last birth before index date*				
< 30 years	7568	48	40,278	50
30-34 years	4815	30	24,172	30
55–39 years	2670	17	12,872	16
40+ years	737	5	3537	4
*Hormonal replacement Therapy (Used at least for 6 months)*				
Estrogen only (50+ years)	2353	22	12,957	31
Continuous progestin (50+ years)	3888	36	12,912	30
Sequential progestin (50+ years)	3092	28	11,566	27
Once in 3 months progestin (50+ years)	350	3	1453	3
E+LNG-IUS (45+ years)	1154	11	3820	9

^a^Controls are classified according to the characteristics of the respective case

Tables 2 and 3 present the association of SES and occupational physical activity among pre- and postmenopausal women, respectively. Significantly increased risk of ductal and lobular breast cancer was observed among both pre- and postmenopausal women for white-collar employees as compared to manual workers. As compared to sedentary occupations, moderate physical activity at work was statistically protective for lobular subtype (OR 0.86, 95% CI 0.78–0.94) among postmenopausal women, while the same OR for premenopausal women remained insignificant (OR 0.86, 95% CI 0.65–1.14). Both pre- and postmenopausal women classified as economically inactive had significant protection against breast cancer risk as compared to sedentary workers.

**Table 2:** Multivariate conditional logistics regression analysis for socio-economic status and occupational physical activity as predictor of breast cancer by histology among women diagnosed in age < 50 years

Variables	Total			Ductal			Lobular		
	*N*	OR	95% CI	*N*	OR	95% CI	*N*	OR	95% CI
*Socio-economic status*									
Manual workers	488	1	Ref	416	1	Ref	46	1	Ref
Lower-level employees	1548	1.20	1.07–1.34	1312	1.17	1.04–1.32	163	1.38	0.96–1.97
Upper-level employees	799	1.35	1.19–1.53	671	1.32	1.15–1.51	91	1.65	1.12–2.44
Others	539	1.00	0.87–1.14	439	0.93	0.81–1.08	63	1.27	0.84–1.92
*Occupational physical activity*									
Sedentary	2128	1	Ref	1790	1	Ref	237	1	Ref
Moderate	674	0.95	0.86–1.05	560	0.96	0.86–1.07	81	0.86	0.65–1.14
High	93	0.99	0.79–1.25	75	0.97	0.75–1.26	13	0.99	0.53–1.82
Economically inactive	493	0.77	0.67–0.88	423	0.77	0.67–0.89	34	0.76	0.48–1.18

Adjusted for parity

*N* number of cancer cases, *OR* odds ratio, *95%CI* 95% confidence interval

**Table 3:** Multivariate conditional logistics regression analysis for socio-economic status and occupational physical activity as predictor of breast cancer by histology among women diagnosed in age 50+ years

Variables	Total			Ductal			Lobular		
	*N*	OR	95% CI	*N*	OR	95% CI	*N*	OR	95% CI
*Socio-economic status*									
Manual workers	2727	1	Ref	2134	1	Ref	448	1	Ref
Lower-level employees	5941	1.12	1.06–1.17	4536	1.10	1.04–1.16	1060	1.15	1.01–1.30
Upper-level employees	2682	1.31	1.23–1.39	2040	1.27	1.19–1.36	490	1.40	1.21–1.62
Others	4461	1.04	0.98–1.10	3435	1.04	0.98–1.11	724	1.03	0.90–1.17
*Occupational physical activity*									
Sedentary	7332	1	Ref	5585	1	Ref	1321	1	Ref
Moderate	6249	0.95	0.91–0.98	4848	0.97	0.93–1.01	1037	0.86	0.78–0.94
High	1755	0.98	0.93–1.05	1347	1.00	0.94–1.07	296	0.93	0.80–1.08
Economically inactive	529	0.76	0.69–0.85	403	0.77	0.69–0.87	76	0.65	0.49–0.85

Adjusted for parity and hormonal replacement therapy use

*N* number of cancer cases, *OR* odds ratio, *95%CI* 95% confidence interval

**Fig. 1:**
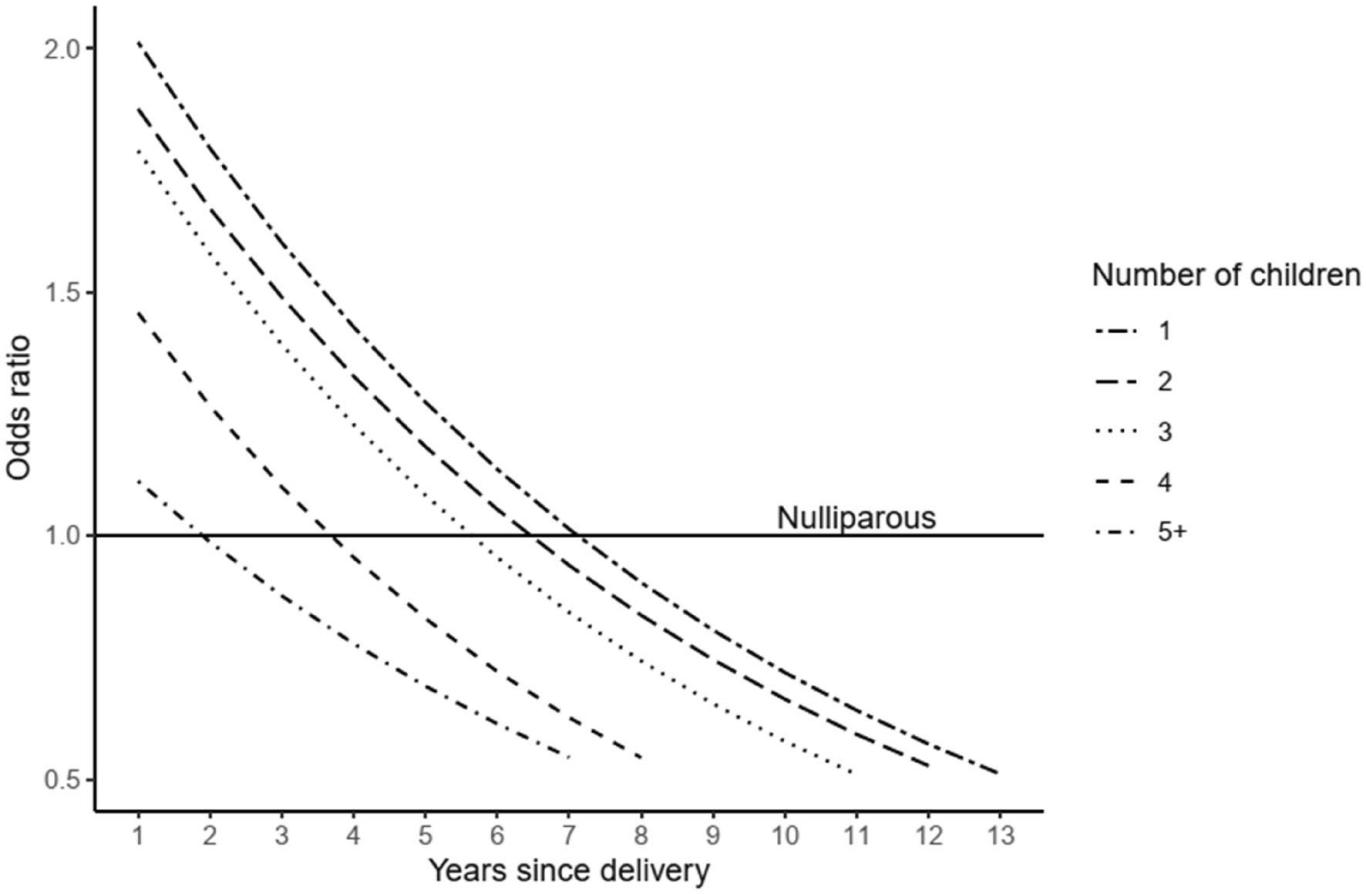
Odds ratios of breast cancer among women diagnosed < 50 years for parous women in comparison to same-aged nulliparous women, by parity and time since the delivery. Multivariate conditional logistics regression analysis Adjusted for socio-economic status

Parous women had 7% reduced risk of premenopausal and 17% reduced risk of postmenopausal breast cancer as compared to nulliparous women (Tables 4 and 5). Among parous women, the increasing number of children was strongly associated with decreasing breast cancer risk (Tables 4 and 5). However, there was a transient increase in the risk for several years after each birth before the incidence decreased below the level of nulliparous women (the model for premenopausal women illustrated in Fig. 1). The peak of transiently increased risk became lower and its duration shorter along with increasing number of children. The model for postmenopausal women is similar as Fig. 1 but with less elevated ORs for the first year after the birth. Increasing age at first and last birth was associated with increased risk of lobular breast cancer among both pre- and postmenopausal women (Tables 4 and 5). For ductal breast cancer, the ages at first and last birth had weaker associations with risk.

**Table 4:** Multivariate conditional logistics regression analysis for reproductive factors as predictor of breast cancer by histology among women diagnosed < 50 years

Variables	Total			Ductal				Lobular	
	*N*	OR	95% CI	*N*	OR	95% CI	*N*	OR	95% CI
*Parity*^a^									
Nulliparous	753	1	Ref	628	1	Ref	77	1	Ref
Parous	2635	0.93	0.85–1.02	2220	0.96	0.87–1.06	288	0.89	0.66–1.19
*Number of children*^b^									
1	620	1	Ref	523	1	Ref	52	1	Ref
2	1223	0.92	0.82–1.04	1022	0.92	0.81–1.05	147	1.05	0.72–1.52
3	588	0.81	0.70–0.95	503	0.84	0.71–0.99	64	0.75	0.46–1.21
4	135	0.64	0.50–0.80	110	0.61	0.47–0.79	19	0.79	0.39–1.59
5 +	69	0.63	0.46–0.86	62	0.69	0.49–0.96	6	0.39	0.14–1.06
*Age at first birth*^b^									
< 20 years	176	1	Ref	157	1	Ref	12	1	Ref
20–24 years	661	1.06	0.88–1.28	573	1.05	0.86–1.28	65	1.37	0.71–2.66
25–29 years	977	1.15	0.95–1.39	806	1.06	0.86–1.30	119	2.02	1.04–3.90
30+ years	821	1.25	0.99–1.57	684	1.20	0.93–1.53	92	1.57	0.74–3.31
*Age at last birth*^b^									
< 30 years	907	1	Ref	780	1	Ref	80	1	Ref
30–34 years	990	1.10	0.97–1.25	835	1.08	0.94–1.23	102	1.33	0.89–1.98
35–39 years	608	1.21	1.03–1.42	498	1.16	0.97–1.38	85	1.92	1.19–3.12
40+ years	130	1.18	0.93–1.50	107	1.12	0.86–1.46	21	2.64	1.33–5.24

Adjusted for socio-economic status, occupational physical activity

*N* number of cancer cases, *OR* odds ratio, *95%CI* 95% confidence interval

^a^The model only included parity

^b^The model included number of children, and age at first and last birth

**Table 5:** Multivariate conditional logistics regression analysis for reproductive factors as predictor of breast cancer by histology among women diagnosed 50+ years

Variables	Total			Ductal				Lobular	
	*N*	OR	95% CI	*N*	OR	95% CI	*N*	OR	95% CI
*Parity*^a^									
Nulliparous	2710	1	Ref	2084	1	Ref	436	1	Ref
Parous	13,155	0.83	0.79–0.87	10,099	0.81	0.77–0.86	2294	0.92	0.82–1.04
*Number of children*^b^									
1	3320	1	Ref	2539	1	Ref	568	1	Ref
2	6094	0.89	0.85–0.94	4669	0.88	0.83–0.93	1100	0.97	0.85–1.10
3	2704	0.79	0.74–0.85	2107	0.79	0.73–0.86	441	0.79	0.67–0.94
4	750	0.71	0.64–0.78	568	0.68	0.61–0.77	134	0.81	0.63–1.03
5 +	287	0.60	0.52–0.70	216	0.59	0.50–0.70	51	0.59	0.41–0.84
*Age at first birth*^b^									
< 20 years	1752	1	Ref	1385	1	Ref	251	1	Ref
20–24 years	5384	0.93	0.88–0.99	4225	0.92	0.86–0.99	843	1.02	0.87–1.20
25–29 years	3842	0.97	0.91–1.04	2899	0.92	0.85–1.00	744	1.29	1.08–1.54
30+ years	2177	0.98	0.89–1.08	1590	0.91	0.81–1.01	456	1.34	1.07–1.70
*Age at last birth*^b^									
< 30 years	6661	1	Ref	5209	1	Ref	1049	1	Ref
30–34 years	3825	1.08	1.02–1.14	2920	1.07	1.01–1.14	689	1.12	0.97–1.28
35–39 years	2062	1.13	1.05–1.22	1521	1.09	1.00–1.19	420	1.35	1.13–1.60
40+ years	607	1.24	1.11–1.38	449	1.19	1.05–1.35	136	1.59	1.24–2.04

Adjusted for socio-economic status, occupational physical activity and hormonal replacement therapy use

*N* number of cancer cases, *OR* odds ratio, *95% CI* 95% confidence interval

^a^The model only included parity

^b^The model included number of children, and age at first and last birth

Table 6 provides ORs for different types of postmenopausal HT therapy to breast cancer risk subtypes. Use of E only therapy for 5+ years significantly increased the risk of ductal (OR 1.17, 95% CI 1.08–1.27) and lobular (OR 1.18, 95% CI 1.00–1.40) breast cancer as compared to non-users. Continuous EPT increased the risk of both breast cancer subtypes, more strongly for lobular subtype and the strength of the association was directly proportional to duration of use. The use of EPT with monthly progestin for 1–< 5 years and 5+ years significantly increased the risk of breast cancer by 1.18 and 1.32-fold, respectively. EPT with progestin once in 3 months was not significantly associated with breast cancer risk. Use of one E+LNG-IUS device (typical use for 5 years) was associated with 1.56-fold increase in the risk of breast cancer (95% CI 1.45–1.69) as compared to never users, while use of more than one device was associated with 2.18-fold increase in risk (95% CI 1.81–2.64). The effect of E+LNG-IUS was similar for both ductal and lobular breast cancer.

**Table 6:** Multivariate conditional logistics regression analysis for postmenopausal hormonal replacement therapy as predictor of breast cancer by histology among women diagnosed 50+ years

Variables	Total			Ductal				Lobular	
	*N*	OR	95% CI	*N*	OR	95% CI	*N*	OR	95% CI
*Estrogen only*									
< 1 year	212	0.95	0.82–1.10	165	0.98	0.83–1.15	34	0.91	0.63–1.31
1–< 5 years	995	1.01	0.94–1.09	801	1.05	0.97–1.14	137	0.86	0.71–1.04
5+ years	1146	1.18	1.10–1.27	875	1.17	1.08–1.27	185	1.18	1.00–1.40
*Continuous progestin*									
< 1 year	347	1.15	1.02–1.30	272	1.18	1.03–1.35	66	1.32	1.00–1.74
1–< 5 years	1947	1.37	1.29–1.45	1476	1.34	1.25–1.43	363	1.57	1.37–1.81
5+ years	1594	2.26	2.12–2.42	1151	2.12	1.97–2.30	374	3.34	2.87–3.90
*Monthly progestin*									
< 1 year	398	1.08	0.96–1.21	280	0.98	0.86–1.11	92	1.46	1.14–1.88
1–< 5 years	2047	1.18	1.12–1.25	1568	1.20	1.13–1.29	383	1.11	0.97–1.28
5+ years	647	1.32	1.20–1.45	485	1.35	1.21–1.51	121	1.14	0.91–1.42
*Once in 3-months progestin*									
< 1 year	65	0.84	0.64–1.10	45	0.78	0.57–1.08	16	0.94	0.53–1.66
1–< 5 years	209	1.00	0.85–1.17	164	1.08	0.91–1.29	32	0.69	0.47–1.03
5+ years	76	0.91	0.71–1.17	50	0.84	0.61–1.14	21	1.19	0.71–1.99
*E*+*LNG-IUS*									
1 purchase	974	1.56	1.45–1.69	762	1.56	1.43–1.70	189	1.59	1.33–1.90
2+ purchases	159	2.18	1.81–2.64	114	2.12	1.70–2.64	33	2.35	1.55–3.58

Adjusted for occupational physical activity, socioeconomic status and parity

*N* number of cancer cases, *OR* odds ratio, *95% CI* 95% confidence interval

## Discussion

Our findings showed that increasing age at first and last birth was associated with increased risk of breast cancer. Higher parity had a protective effect on the breast cancer but there was a transient increase in the risk after each pregnancy lasting for several years. Women in white-collar work and lower level of occupational physical activity had increased risk of breast cancer. Long-term use of EPT and E+LNG-IUS hormone therapy contributed strongly to the excess risk of breast cancer incidence.

### Socioeconomic status

White-collar employees had higher risk of both pre- and postmenopausal breast cancer than blue-collar workers despite that our results were adjusted for parity and occupational physical activity and also for HT among the postmenopausal women. Most of the previous studies have shown similar association between different measures of increasing SES and breast cancer risk [20–22, 29, 30]. These studies however normally were not able to separate the effect of longer education and career planning leading to postponing of childbirth and resulting in lower parity and, the effects of other lifestyle factor in women with higher SES such as alcohol consumption and dietary habits nor take into account the greater access and use of exogenous hormones which may have been more is common among women in higher SES and increase their risk of postmenopausal breast cancer.

A previous study in Finland showed that age at first birth and average number of children did not vary by SES [31]. However, the non-modifiable reproductive risk factors such as early age at menarche and late age at menopause were observed among women with higher SES in Finland [32]. Therefore, these physiological differences and other aspects of SES such as alcohol consumption, which is more common among women with higher SES group in Finland might have been important risk contributor [33]. This might also explain that the greater strength of associations observed in our study for lobular breast cancer as compared to ductal subtypes in the upper-level employees, as lobular breast cancer is more sensitive to factors, such as alcohol consumption, that cause alterations in hormonal status.

### Occupational physical activity

In our study, increased occupational physical activity was associated with lower breast cancer risk with the modest protective effect observed for lobular subtype and even smaller effect for ductal subtype. The smaller effect of physical activity in our study as compared to some earlier studies could be because our findings are adjusted for reproductive history and use of HT. We observed that the risk of breast cancer in both pre- and postmenopausal group was significantly reduced among the women who were economically inactive. Large proportion of this category comprised of spouses of farmers who are physically very active. Studies on association of breast cancer and physical activity have produced inconsistent results. While most studies show that occupational sedentariness is associated with increased risk of breast cancer [16, 34–38], few studies have shown borderline to no association [39–41]. Physical activity reduces the levels of circulating sex steroids that increases the risk of breast cancer among pre- and postmenopausal women [42–44]. Other mechanisms of breast cancer risk reduction through physical activity are via reduction in fat, boosting of immune system, and decreased insulin levels in body [42].

### Reproductive factors

Increasing age at first birth was in our study associated with increased risk of lobular but not ductal breast cancer, similarly among pre- and postmenopausal women. Stronger association of the higher age at first birth with lobular breast cancer than with ductal subtypes was also observed in a large previous meta-analysis [45] and suggested in other studies [46–50]. First pregnancy exhibits maximum cellular differentiation and maturation of breast cells making them more resistant to carcinogenic effects [51, 52]. Increasing age at first birth and thereby longer duration between menarche and first birth, during which the undifferentiated breast tissue is subjected to tumor promoting effects of ovarian hormones in each menstrual cycle [50, 53]. Since lobular cancer is almost always estrogen receptor-positive and consequently more hormone sensitive than ductal cancer [54], an increase in the risk of lobular breast cancer with increasing age at first birth was expected.

Like previous studies [55–59], the present results showed that increasing parity is associated with lowered breast cancer risk. Furthermore, our results are well in line with earlier findings stating that pregnancy has dual effect on breast cancer risk, i.e., a long-term protective effect of a pregnancy is preceded by a transient increase of the risk after birth that can last up to 3–15 years [52, 60, 61]. Our findings among premenopausal women (Fig. 1) showed that women who had one child as compared to nulliparous women showed two-fold increased risk of breast cancer immediately after birth and the risk gradually decreased with protective effect observed about 7–8 years after first child. With each additional birth, the transient increase in risk was observed, however, the peak was smaller than in the previous birth. After fifth birth, there was only less than 20% transient excess risk as compared to nulliparous women, and protective effect started much earlier, about two years after the childbirth. Similar pattern of dual effect was seen for postmenopausal women but with a smaller transient increase in risk of breast cancer immediately after birth. We chose to present the findings for premenopausal breast cancer only because great majority of the postmenopausal breast cancers are diagnosed more than 10 years after childbirth and the modeling of the risk immediately after the birth is less strong. In a model combining both pre- and postmenopausal women, the peak of transient increase in the first-year peak was lower (OR about 1.5 after the first and second birth and below 1.0 for women after 4+ birth).

There is continuous increase of estrogens and other steroids during pregnancy, which are important for mammary gland development [9, 62, 63]. Consequently, they may play a role in proliferation and neoplastic transformation of breast cells [10, 63, 64] and cause transient increase in the risk after birth lasting for several years before protective effect is observed [51, 52].

### Postmenopausal hormone therapy

Estrogen-only therapy was associated with elevated risk for both ductal and lobular cancers in long-term use of more than 5 years, but the magnitude of the effect was small compared to combined therapy. The findings from a recent meta-analysis of worldwide data showed that the effect of estrogen in breast cancer risk was doubled when the use was increased from 1 to 4 years to 5+ years, with 33% excess risk among estrogen-only users as compared with non-users [65]. Other previous studies suggest that the effect of estrogen-only HT is small and cannot be detected in short-term follow-up [66–68]. In Finland, previous studies have consistently shown that estradiol is associated with a moderate increase in breast cancer risk [11, 66].

Estrogen combined with progestin was more strongly associated with breast cancer than the use of estrogen-only therapy in our study, which has been shown also in previous studies [65, 69–71]. Addition of a progestin to estrogen therapy enhances the breast cells proliferation and number of cells present in terminal ductal lobular units increasing the risk of malignant transformation of breast tissues [72, 73]. Furthermore, the continuous use of progestin combined with estrogen was associated with higher relative risk than cyclic progestin used once a month or once in three months. This observation is also in line with previous studies [66, 71, 74]. Additionally, among continuous progestin therapy users, the risk of breast cancer increased linearly with duration of use and the relative risk was higher for lobular as compared to ductal cancer. Lobular breast cancer is hormonally more sensitive and continuous use of the combined EPT even for a short duration may cause significant proliferation of lobular cells resulting in cancer [69, 75].

We observed a strongly increased risk of both ductal and lobular breast cancer among E+LNG-IUS users. The magnitude of risk was higher for women who used more than one LNG-IUS device. A recent meta-analysis concluded an increased risk of breast cancer among LNG-IUS users regardless of age, and larger increase in risk was observed among older women [76]. The greater incidence of breast cancer among LNG-IUS users was observed also in some previous Finnish studies [77, 78].

## Strengths and limitations

Our study has several strengths. The study is based on large number of breast cancer cases registered by the Finnish Cancer Registry, which is virtually complete as regard to cancer incidence since 1953 [79, 80], meaning that our results are strictly population-representative without selection bias. The Finnish Population Registry includes accurate information on childbirths of women born after the mid-1930s. Information on study variables had been registered in high-quality population-based registries similarly for cases and controls and we therefore do not have recall bias. We were able to include in the same model several important components of breast cancer etiology including reproductive history, SES, and occupational physical activity, and HT use, and hence to assess their independent roles in breast cancer etiology. However, the study has some limitations as well. A major limitation of our study is that we did not have information on estrogen receptor (ER) status of the breast cancer and were unable to distinguish the risk between ER positive and ER negative breast cancer. We did not have information on the family history of the breast cancer of cases and controls. Similarly, information such as age at menarche, age at menopause, body mass index (BMI), and history of breastfeeding among parous women were not available. Among Finnish female population, BMI is lower for higher SES and vice versa [81]. Therefore, we would expect to get even higher risk estimate for high SES if we would have been able to adjust the OR for the BMI.

## Conclusion

Our study confirms that increasing age at first and last birth increases risk of breast cancer while increasing parity has a protective effect, but the effect is not straightforward: the long-term protective effect of parity is preceded by a transient increase in the risk after each pregnancy, which lasts for several years. We found that the selection of HT type affects the breast cancer incidence among postmenopausal women, and this effect remains after adjustment for parity and SES. Long-term estrogen treatment with continuous progestin or LNG-IUS was strongly associated with breast cancer risk irrespective of the histology of the cancer. Women with higher SES had elevated risk of breast cancer while occupational sedentariness only carried a minor excess after adjustment for parity and HT. Sedentary work is an important risk factor of BC from public health point of view because of large and increasing fraction of women in sedentary occupations. Multivariate setting with several factors of BC potentially associated with women’s reproductive, socio-economic and lifestyle selection is necessary to understand the true influences of these factors.

## Supplementary Information

Below is the link to the electronic supplementary material.

Supplementary file1 (PDF 40 kb)

## Data Availability

Enquiries about data availability should be directed to the authors.

## Abbreviations

CI: Confidence interval
E: Estradiol
EPT: Estradiol Progestin Therapy
E+LNG-IUS: Estradiol (oral or transdermal) plus levonorgestrel-releasing intrauterine system
HT: Hormonal therapy
ICD: International Classification of Diseases
ISCED: International Standard Classification of Education
ISCO: International Standard Classification of Occupations
NOCCA: Nordic Occupational Cancer Study
NOCCA-JEM: Job Exposure Matrix of the NOCCA study
OR: Odds ratio
PPWL: Perceived physical workload
SES: Socio-economic status
