# Supplementary material for: Multivariate analysis of independent roles of socioeconomic status, occupational physical activity, reproductive factors, and postmenopausal hormonal therapy in risk of breast cancer
Source: Breast Cancer Res Treat. 2022 Apr 2;193(2):495–505. doi: 10.1007/s10549-022-06571-x (PMC9090885; doi:10.1007/s10549-022-06571-x)
Supplement: Supplementary file 1 — Supplementary file1 (PDF 40 kb) [file 10549_2022_6571_MOESM1_ESM.pdf]

Classification of socio-economic status 1989

Code	Item name	Category in this study
**1**	**Self-employed persons**	**Others**
11	Farmer etc. employers	
12	Farmers etc. on own account	
21	Small employers	
22	Other employers	
23	Own-account workers	
24	Self-employed persons in liberal professions	
29	Self-employed persons, unspecified	
**3**	**Upper-level employees with administrative, managerial, professional and related occupations**	**Upper-level white-collar employees**
31	Senior officials and upper management	
32	Senior officials and employees in research and planning	
33	Senior officials and employees in education and training	
34	Other senior officials and employees	
39	Senior officials and employees, unspecified	
**4**	**Lower-level employees with administrative and clerical occupations**	**Lower-level white-collar employees**
41	Supervisors	
42	Clerical and sales workers, independent work	
43	Clerical and sales workers, routine work	
44	Other lower-level employees with administrative and clerical occupations	
49	Lower-level employees, unspecified	
**5**	**Manual workers**	**Manual workers**
51	Workers in agriculture, forestry and commercial fishing	
52	Manufacturing workers	
53	Other production workers	
54	Distribution and service workers	
59	Workers, unspecified	

**6**	**Students**	**Others**
**7**	**Pensioners**	**Others**
71	Retired self-employed persons	
72	Retired upper-level employees	
73	Retired lower-level employees	
74	Retired manual workers	
79	Other pensioners	
**8**	**Others**	**Others**
81	Long-term unemployed	
82	Others not elsewhere classified	
99	Socio-economic status unknown	
